# Long-term in vitro recording of cardiac action potentials on microelectrode arrays for chronic cardiotoxicity assessment

**DOI:** 10.1007/s00204-022-03422-y

**Published:** 2023-01-06

**Authors:** Giuseppina Iachetta, Giovanni Melle, Nicolò Colistra, Francesco Tantussi, Francesco De Angelis, Michele Dipalo

**Affiliations:** 1 Istituto Italiano di Tecnologia, Via Morego 30, 16163 Genova, Italy; 2 FORESEE Biosystems Srl, Genova, Italy

**Keywords:** Chronic cardiotoxicity, Optoporation, Intracellular recording, Cardiomyocytes, In vitro toxicology, Action potentials

## Abstract

The reliable identification of chronic cardiotoxic effects in in vitro screenings is fundamental for filtering out toxic molecular entities before in vivo animal experimentation and clinical trials. Present techniques such as patch-clamp, voltage indicators, and standard microelectrode arrays do not offer at the same time high sensitivity for measuring transmembrane ion currents and low-invasiveness for monitoring cells over long time. Here, we show that optoporation applied to microelectrode arrays enables measuring action potentials from human-derived cardiac syncytia for more than 1 continuous month and provides reliable data on chronic cardiotoxic effects caused by known compounds such as pentamidine. The technique has high potential for detecting chronic cardiotoxicity in the early phases of drug development.

**Supplementary Information:**

The online version contains supplementary material available at 10.1007/s00204-022-03422-y.

## Introduction

The process of drug discovery is extremely long and expensive, with cost estimates ranging from $314 million to $2.8 billion and a period of 10–15 years to commercialize a new drug (Morgan et al. 2011; DiMasi et al. 2016). Considering the development cost and time, the reduction of high attrition rates during this process is still a key challenge for the pharmaceutical companies (Hay et al. 2014). Although reasons for drug attrition and market withdrawals are several, the major cause of drug failure can be associated with their cardiac adverse effects (Ferdinandy et al. 2019).

The current cardiac safety pharmacology guidelines (ICH S7B and E14) mandate in vitro measurements of the human-ether-à-go-go related gene (hERG) potassium channel activity followed by in vivo QT prolongation measurements as traditional standards for preclinical screening assay to evaluate proarrhythmic risk of all candidates drug (FDA 2005a, b). In addition, the Comprehensive in vitro Proarrhythmia Assay (CiPA) initiative, established in 2013, and the Japan iPS Cardiac Safety Assessment (JiCSA) established in 2014 to develop a new paradigm for assessing proarrhythmic risk, validated the use of microelectrode array (MEA) and cardiomyocytes derived from human induced pluripotent stem cells (hiPSC-CMs) to assess drug induced cardiotoxicity during the in vitro preclinical phase (Colatsky et al. 2016; Kanda et al. 2016; Kitaguchi et al. 2016; Takasuna et al. 2017; Blinova et al. 2018). In parallel, research groups are evaluating the implementation of 3D nanostructures on MEAs and the development of novel optical approaches to enable recordings of cardiac action potentials (APs) from in vitro two-dimensional syncytia (Dipalo et al. 2017; Desbiolles et al. 2020; Hu et al. 2020; Barbaglia et al. 2021; Zhou et al. 2021; Jahed et al. 2022). However, current methods for in vitro cardiotoxicity screenings such as patch-clamp, voltage indicators, and standard MEAs do not offer at the same time high sensitivity for measuring transmembrane ion currents and low-invasiveness for monitoring cells over long time. Patch-clamp permits to assess the drug effects on cardiac ion channels and to evaluate the transcellular cardiac action potential with high spatial and temporal resolution (Rampe et al. 1997; Kramer et al. 2013), but it is limited to only single cells and it cannot be applied for drug chronic effects due to its invasiveness. Voltage-indicators allow non-invasive assessment of both single cell and syncytium activity but their cytotoxic effects make difficult the application for long-term assessment (Herron et al. 2012; Ronzhina et al. 2021).

Other methods like standard MEAs and impedance monitoring provide non-invasive and long-term measurements of electrical signals from electrogenic cells. However, these technologies are limited to record extracellular field potentials (FPs) instead of the intracellular action potentials (APs) (Stett et al. 2003; Poulton 2017; Tertoolen et al. 2018), lacking crucial parameters such as the difference between FP prolongation and triangulation (Hondeghem and Hoffmann 2003; Deo et al. 2020) and other minor proarrhythmic events (e.g. early and delayed afterdepolarizations, EADs and DADs). Notably, several compounds such as hERG channel trafficking inhibitors (e.g. pentamidine) (Dennis et al. 2007; Obergrussberger et al. 2016; Asahi et al. 2019) and chemotherapeutic molecules (e.g. doxorubicin) (Kumar et al. 2012; Chaudhari et al. 2017; Mladěnka et al. 2018; Narezkina and Nasim 2019; Bozza et al. 2021) may not show their cardiotoxic effects after acute dose administration but may become toxic after repeated and prolonged exposures (several hours to days) (Cai et al. 2019). Thus, adverse cardiac effects occurring after long-term drug exposure may be undetected during the preclinical screening and may allow for the further development of potentially toxic molecules. Although hiPSC-CMs can be maintained in culture for long periods of time with stable baseline functions (Kopljar et al. 2017; Dias et al. 2018), the current methods such as patch-clamp, voltage indicators, and MEA technology do not offer at the same time high sensitivity for measuring transmembrane ion currents and low-invasiveness for monitoring cells over long time. Therefore, despite the recent advances in chronic cardiotoxicity assays using hiPSC-CMs (Narkar et al. 2022), long-term (chronic) cardiac adverse effects remain a major barrier of drug development. The in vitro detection of chronic cardiotoxicity represents a key turning point to avoid the progression of a drug candidate with “delayed” cardiac adverse effect. Thus, to render the drug development process more efficient, there is an immediate need to develop standardized assays to detect chronic cardiotoxicity during in vitro screenings.

We recently proposed and validated a novel approach for high-quality recordings of intracellular action potentials (APs) simultaneously from large cardiomyocyte networks combining commercial MEA technology with cell optoporation (Dipalo et al. 2018; Melle et al. 2020; Iachetta et al. 2021). Our approach improves the reliability of acute cardiotoxicity drug screenings reducing the cell cultures required to reach exploitable results in cardiac safety assessment.

By exploiting the non-invasiveness of optoporation (Messina et al. 2015) and a new experimental procedure based on backside laser excitation of transparent electrodes, in this work we demonstrate that it enables extremely long-term AP recordings of the same cells on commercial MEAs, paving the way to the reliable chronic cardiotoxicity assessment on hiPSC-CMs. We used optoporation on multiwell 60-electrode commercial MEAs with titanium nitride (TiN) transparent and thin electrodes to measure the APs from the same hiPSC-CMs for up to 35 days in vitro in a raw. Such monitoring time windows are almost 10 times longer than what is possible with commercial techniques and more than double than what is shown on alternative cutting-edge technologies (Jahed et al. 2022).

To demonstrate the performances for cardiotoxicity screening, we also measure how a known compound, pentamidine, affects cardiac ion channels over long-term exposure. As expected, the molecule does not affect the AP waveforms after acute administration. However, we can correctly observe the effects on the cardiac cells as exposure time increases up to hundreds of hours and the cardiomyocytes recovery during the subsequent drug washout period. Furthermore, given the importance of chronic cardiotoxicity in the development of new anticancer drugs, we further tested our approach by monitoring the effects of repeated administration of doxorubicin on the electrophysiological activity of hiPSC-CMs for an extremely long period. Finally, we demonstrate that the long-term assessment of APs by consecutive optoporation sessions has no effects on cell health.

Our results demonstrate that optoporation may be a valuable tool to develop reliable cardiac cellular models for therapeutic and diagnostic applications and to assess the delayed drug-induced cardiotoxicity during preclinical phases of drug development process.

## Materials and methods

### hiPSC cardiomyocytes culture on commercial MEAs

hiPSC-derived cardiomyocytes were purchased from Cellular Dynamics International and Ncardia. iCell cardiomyocytes (Cellular Dynamics International) were directly plating on 60-6wellMEA200/30iR-Ti-rcr (Multichannel Systems) at a density of 16,000 cells per well and grown according to the specifications of the commercial supplier. MCS MEAs were coated with fibronectin (50 μg/ml) for 1 h at 37 °C, 5% CO_2_ in a humidified incubator to promote the cells adhesion. Electrophysiological recordings of iCell cardiomyocytes were performed starting from 9 days post-plating and repeated every 2–3 days. Cor.4U (Ncardia) were pre-cultured in a cell culture T25 and then seeded at the density of 10,000 cells per well in 60-6wellMEA200/30iR-Ti-rcr (Multichannel Systems). MCS MEAs were previously coated with geltrex ready-to-use solution (ThermoFisher) for 30 min at 37 °C, 5% CO_2_ in a humidified incubator. Electrophysiological recordings of Cor.4U cardiomyocytes were performed starting from 3 days post-plating and repeated every day.

### Chemical compounds

Pentamidine, dofetilide, nifedipine and doxorubicin were purchased from Sigma-Aldrich. The 10 mM stock solutions were prepared in dimethyl sulfoxide (DMSO). Drug dilutions were performed in pre-warmed (37 °C) cardiomyocytes medium. Data of control with DMSO are reported in Supplementary Fig. S2.

### Electrophysiological recording

All recordings from MCS-MEAs were performed at 37 °C outside the incubator. The cell medium from each sample was changed 2 h before the measurements. All data sets were analyzed by Matlab software. MEA recordings were obtained with a MEA2100-Mini-System from the company Multi Channel Systems MCS GmbH. Recordings with the MEA2100-Mini-System were acquired at 20 kHz acquisition frequency, high-pass hardware filtering of 0.1 Hz, and low-pass hardware filtering of 10,000 Hz.

### Immunofluorescence staining and live/dead assay

After six consecutive intracellular recording sessions (21 DIVs), for immunofluorescence staining, the cells were fixed with 4% (w/v) formaldehyde in phosphate-buffered saline (PBS), permeabilized with 1% saponin (15 min), blocked with 3% bovine serum albumin (BSA) for 30 min followed by incubation with TNNT2 (mouse anti-TNNT2 A25969) antibody diluted 1:1000 in a solution of 3% BSA for 3 h at room temperature. After three washes in PBS, cells were incubated with Alexa Fluor 488 donkey anti-mouse secondary antibody (diluted 1:250 in blocking solution) for 1 h at room temperature, followed by three washes with PBS. Finally, cell nuclei were counterstained with DAPI cells and then the images were acquired using an inverted Nikon microscope by using filters for Alexa 488 channel (Ex/Em 495/519 nm) and DAPI channel (Ex/Em 358/461 nm). Cell viability was tested using live/dead assay (ThermoFisher L3224) after six consecutive intracellular recording sessions (21 DIVs). Live cells were stained with green fluorescence (Calcein acetoxymethyl, Calcein AM) whereas dead cells were stained with red fluorescence (Ethidium homodimer-1, Eth-D1). The images were acquired using an inverted microscope (Nikon Eclipse TE2000-E) with a 20× air objective (Plan Fluor 20X N.A. 0,45 LWD inf/0–2 W.d. 8.2–6.9) and filters for FITC channel (ex/em 488/515 nm) and TRITC channel (ex/em 570/602 nm).

### Laser optoporation

Laser pulse trains were applied on the surface of MCS MEAs to porate hiPSC-CMs. For laser poration, the first harmonic (*λ* = 1064 nm) of a Nd:YAG (neodymium:yttrium–aluminium–garnet) solid-state laser (Plecter Duo (Coherent)) with an 8 ps pulse width and 80 MHz repetition rate was used as radiation source, with an average power of approximately 9 mW after the objective. The laser was coupled to an inverted optical setup able to accommodate the acquisition system MEA2100-Mini from Multi Channel Systems MCS Gmbh. A 50× air objective (NA = 0.45, working distance = 25 mm) was used during the experiments to observe the cells on the devices and to focus the NIR laser used for poration.

### Data and statistical analysis

A custom-made algorithm was specifically developed in MATLAB (The Mathworks, Natick, MA, USA) to perform data analysis. The algorithm allows for accurate characterization of the intracellular action potentials waveforms, the intracellular APs duration at different amplitude values with respect to the maximum amplitudes (30, 50 and 90%), and the beating rate. Data were expressed as mean ± standard error of the mean (SEM). Statistical analysis was performed to determine significant differences between the measurement at each DIV and the value at the first DIV of measurement by using MATLAB. Since data do not follow a normal distribution (evaluated by the Kolmogorov–Smirnov normality test), a non-parametric Mann–Whitney *U* test was used. An example of data distribution is reported in Supplementary Fig. S7. Statistical tests were considered significant for *p* < 0.05.

## Results

### Long-term monitoring of APs from hiPSC-CMs

**Fig. 1:**
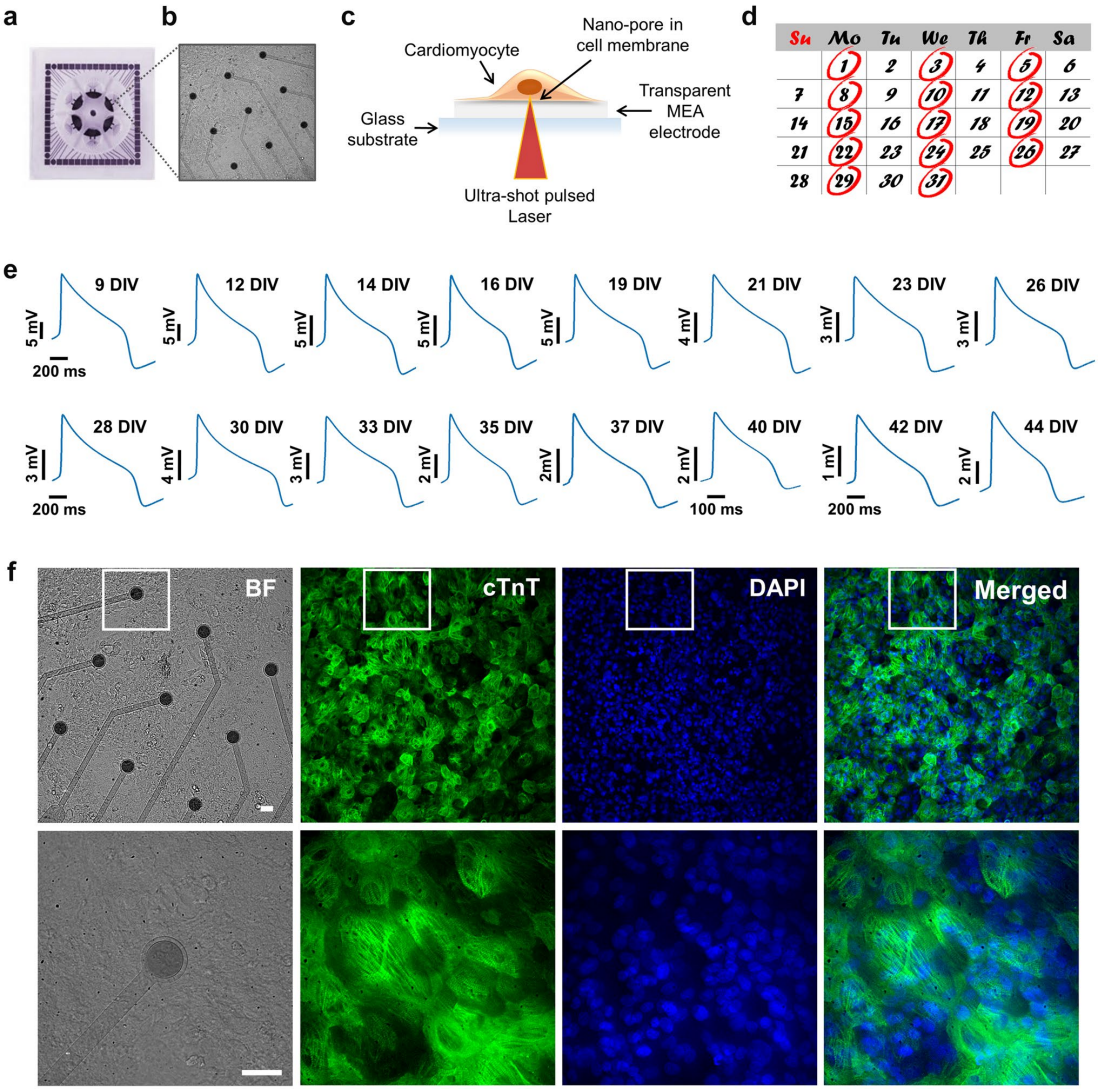
Long-term intracellular AP recordings from hiPSC-CMs. **a** Photograph of a 6-well MEA (60-6wellMEA200/30iR-Ti-rcr). **b** hiPSC-CMs at 9DIVs in a single MEA well. **c** Representative scheme of laser-based optoporation on 6-well MEA devices. **d** Time course of APs recording. **e** Action potential mean waveforms of cardiac syncytium in a well of 6-well MEA at different DIVs. **f** Immunofluorescence images at 20× magnification of hiPSC-CMs on 6-well MEA after 6 repeated opotoporation procedures and at 60× magnification of the area highlighted with white box. Scale bar: 30 µm

We cultured hiPSC-CMs on 60-electrode 6-well commercial MEAs (Fig. 1a, b). The employed commercial MEA presents transparent titanium nitride (TiN) electrodes that allow for applying the laser-based optoporation protocol from the bottom of the device (Fig. 1c) by means of an inverted optical setup. Thanks to the electrode transparency, laser radiation from the bottom transmits through the electrode and reaches the electrode-cell interface, where it generates charge ejection from the electrode material and leads to localized poration of the cellular membrane (Melle et al. 2020). Optoporation from the backside of the MEA devices is fundamental for preserving a perfect sealing of the device during recordings, helping maintaining the necessary sterility for long-term measurements. We performed optoporation and intracellular AP recording starting after 9 days in vitro (DIVs) and we repeated the measurements every 2–3 days up to a maximum of 44 DIVs, for a total of more than 30 monitoring days (Fig. 1d). After this time window, cardiac cells started to show detachment from the MEA surface and measurements became unreliable. In each measurement day, thanks to an automatic poration by means of laser scanning, we recorded intracellular APs from all 9 electrodes in each well of the MEA obtaining the mean AP waveform of each cardiac monolayer. We recorded approximately 3 min of intracellular signals (corresponding to approximately 100 recorded APs). Figure 1e depicts exemplary mean APs at different DIVs from the same cardiac monolayer (the same well in the 6-well MEA). The AP waveforms present high signal-to-noise ratio and allow us for evaluating transmembrane ion currents from the same syncytium over extremely long periods. Moreover, one can also observe a reduction of APs amplitude over time, likely due to the gradual reduction of adhesion of the cardiac cells on the MEA surface because of their contraction (Fig. 1e). Furthermore, to evaluate the effect of repeated optoporation on cardiomyocytes health and distribution of cells on MEA, live/dead assay and immunofluorescence for cardiac troponin-T (cTnT) were performed on hiPSC-CMs after six consecutive intracellular recording sessions. As reported in Supplementary Fig. S1, the cell viability displays a uniform syncytium of healthy cardiomyocytes, demonstrating that repeated intracellular recording procedures have no substantial effects on the cell health. Immunofluorescence images highlights the typical organization and filament-like structure of Troponin T of cardiomyocytes after repeated optoporation, demonstrating that the long-term assessment of APs has no significant effects on the cell health and distribution on MEAs (Fig. 1f). Thus, our approach permits the long-time assessment of APs preserving the health of the cardiac cells.

**Fig. 2:**
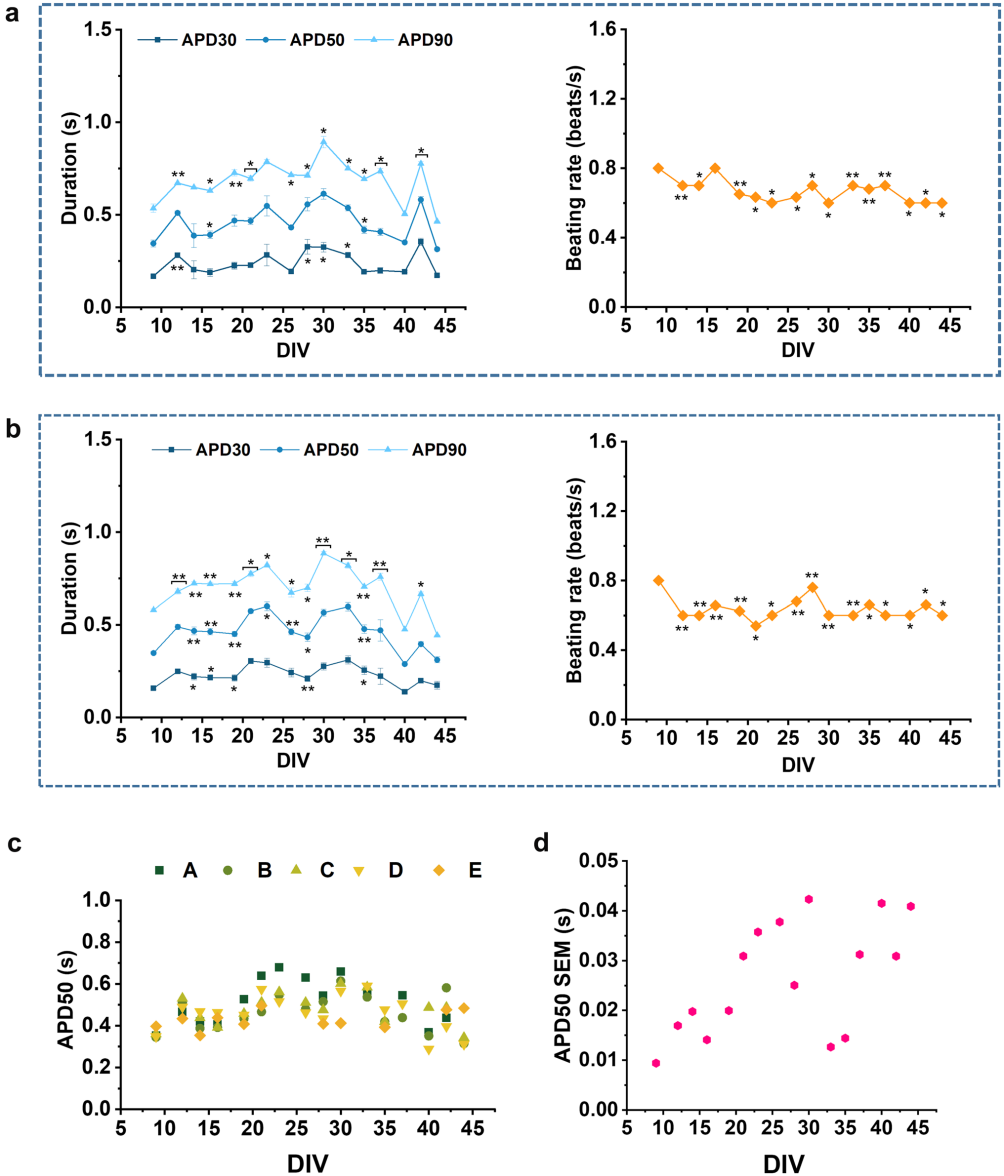
Long-term intracellular AP recordings from hiPSC-CMs. **a, b** Action potential duration at different amplitudes (APD30, 50, 90) and beating rate for two different wells of a 6-well MEA. **c** Comparison of the APD at 50% repolarization (APD50) of each well over time. **d** Standard error of the mean (SEM) of APD50 among wells. Data are represented as mean ± SEM

Furthermore, our analysis of AP duration at different amplitudes (30% (APD30), 50% (APD50) and 90% (APD90)) of repolarization shows variations of APD and beating rate over days. These parameters remain though within a range compatible with data reported in literature for this cell line (Blinova et al. 2017; Schaefer et al. 2022) (Fig. 2a, b). Moreover, considering that cardiomyocytes show significant changes in overall gene expression from day 2 to day 14 post-thaw (Puppala et al. 2013), the long-term evaluation of APs shape and duration using our approach may be useful to better characterize this model and other new cardiac model for drug screening.

In Fig. 2c, we report APD50 values of 5 independent wells in a 6-well MEA of the same cell culture preparation from 9 to 44 DIVs. At 9 DIVs, we observe that APD50 values are relatively similar as it is expected because cells have been treated exactly in the same way and had a short time to diverge. As the cells remain longer in culture, we observe that APD50 values start to diverge among wells. This is highlighted in Fig. 2d where we present the standard error of the mean (SEM) of APD50 among the wells along the whole culture time. It can be observed that SEM tends to increase over time. We suggest this to be an important factor to consider when assessing long-term cardiotoxicity, because the comparison between independent syncytia could lead to wrong conclusions about chronic drug effects. More reliable results can be indeed obtained by testing drugs within the same syncytium, without using a different culture well as control.

### Long-term AP monitoring from single cells

**Fig. 3:**
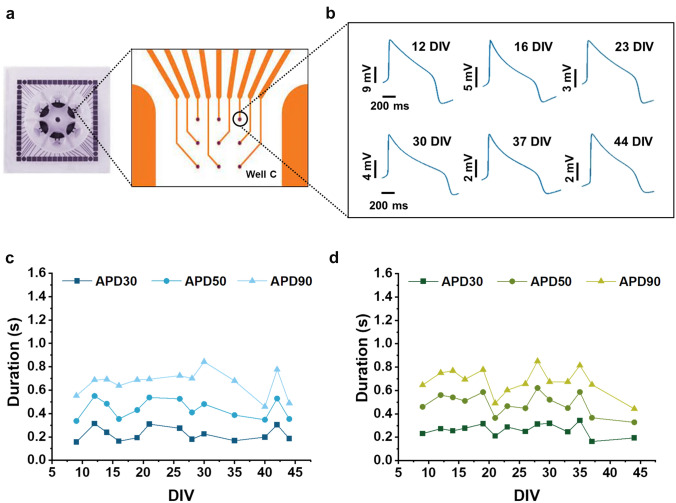
Long-term intracellular AP recordings from single cardiac cells. **a** Photograph of a well of 6-well MEA (60-6wellMEA200/30iR-Ti-rcr). **b** Action potential waveform of single cells in a well of a 6-well MEA at different DIVs. **c, d** Action potential duration at different amplitudes (APD30, 50, 90) from two single electrodes in two different wells

Our approach for long-term AP recordings not only represents a valuable tool for long-term assessment of large hiPSC-CM syncytia, but it could also enable long-time monitoring of APD from single cardiac cells in each well of 60-electrode 6-well commercial MEAs (Fig. 3a). Figure 3b shows AP at different DIVs from the same electrode. Although cardiomyocytes exhibit spontaneous migratory activity in vitro, we can assume a measurement of APs from the same cell for short time intervals within the whole long-term recording experiment. The AP duration analysis at different amplitudes (APD30, APD50 and APD90) of repolarization from two single electrodes in two different wells highlights the variations of APD over days (Fig. 3c, d). Such feature results from the extremely low invasiveness of cell optoporation, which allows for porating and measuring the same cell for several times without negative effects.

The monitoring of changes in cardiac transmembrane ion currents of single cells in large syncytia is fundamental for evaluating cell-specific drug effects. Indeed, inhomogeneous drug distribution (Pinto and Howell 2007) can influence the electrical response of cells to the same drug.

Furthermore, despite the progresses in development of subtype-specific differentiation protocols (Zhang et al. 2011; Devalla et al. 2015; Birket et al. 2015), obtaining pure populations of ventricular-, atrial-, or nodal-like hiPSC-CMs is still challenging. Following the AP evolution of single cardiac cells may help the electrophysiological classification of hiPSC-CMs into cardiac subtypes based on their AP profiles, as this is currently the gold standard for cardiomyocytes characterization (Zhang et al. 2009; Lieu et al. 2013). However, the existing approaches to assess the AP profiles make the cells unviable for subsequent analysis preventing the proper monitoring of maturation of the cardiomyocytes over time. Our approach may overcome this limitation enabling the monitoring in time of hiPSC-CM maturation, facilitating the development of more reliable and predictive cardiac cellular models intended for therapeutic and diagnostic applications. Moreover, several groups are working on methods for immobilizing cardiomyocytes on substrates such as glass (Davis et al. 2021). In perspective, the combination of such approaches with our technology may enable the monitoring of single cells over long period of time.

### Chronic drug effects on cardiac ion channels

To assess the ability of our approach to detect long-term cardiotoxic drug effects, we tested a hERG trafficking inhibitor, pentamidine, which produces adverse cardiac effect after several hours of continuous exposure (Asahi et al. 2019). Pentamidine is used to treat several parasitic diseases but it is also associated with drug-induced long QT syndrome and Torsades de Pointes (TdP) (Obergrussberger et al. 2016). Interestingly, this drug has no direct hERG-blocking activity but inhibits the hERG trafficking to the cell membrane, reducing its expression on the cell surface (Cordes et al. 2005; Nogawa and Kawai 2014). This mechanism of action may go undetected in most conventional cardiac safety assays (i.e. conventional or automated patch clamp), highlighting the necessity for longer-term assays to evaluate indirect effects on the ion channels.

**Fig. 4:**
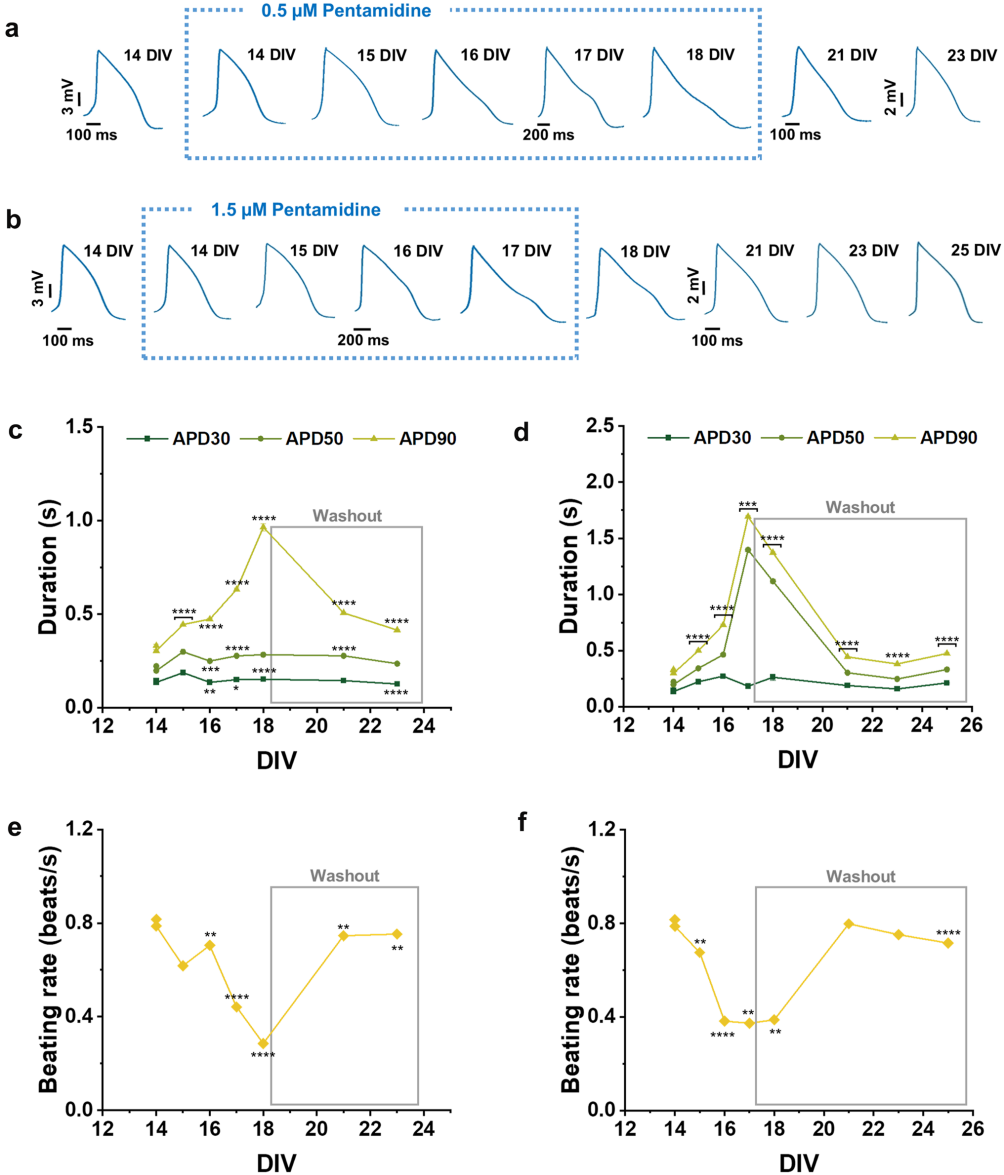
Long-term effect of pentamidine on hiPSC-CMs. **a, b** Action potential mean waveforms at different days after 0.5 and 1.5 µM pentamidine administration, respectively. **c, d** Action potential duration at different amplitudes (APD30, 50, 90) after 0.5 and 1.5 µM pentamidine administration, respectively. **e, f** Beating rate after 0.5 and 1.5 µM pentamidine administration, respectively. Data are represented as mean ± SEM of 3 wells

We monitored APs of the same hiPSC syncytia up to 11 DIVs (from 14 DIVs to 23 and 25 DIVs), exposing cells to pentamidine for 3–4 DIVs and then monitoring cell recovery after drug washout. We tested pentamidine at 0.5 and 1.5 µM concentrations. Figure 4a, b depicts AP waveforms along the whole 11 DIVs experiment at the two different concentrations. In Fig. 4c and 4d, we summarize the effects by tracing APD30, APD50 and AP90 over time in 3 wells. Pentamidine shows both concentration- and time-dependent effects after long exposure time. At both tested concentrations, pentamidine leads to APD increase starting from 24 h of incubation and is strengthened in the following incubation days (Fig. 4c, d). At higher concentration of pentamidine, the AP elongation is greater and occurs earlier compared to the lower concentration. Indeed, the effect can still be observed 1 day after drug washout (Fig. 4d). In some cases, we also detect early afterdepolarization events (EADs), abnormal depolarizations during the repolarization phase of AP associated with arrhythmias (Weiss et al. 2010; Qu et al. 2013), for both concentration of pentamidine (Supplementary Fig. S3). Furthermore, we checked whether the cardiac monolayer had the ability to spontaneously recover from pentamidine effect by monitoring the APD after the drug washout. Our results show a return to the APD physiological range for both tested concentration, this occurs the day after drug washout for the lower concentration and two days after drug washout for the higher concentration of pentamidine administrated (Fig. 4c, d). For comprehensiveness, we also report in Supplementary Fig. S4 the APD90 data expressed as percentage of variation in respect to the control. Finally, we observe also beating rate reduction induced by pentamidine in a concentration-dependent manner (Fig. 4e, f).

Furthermore, considering that hiPSC cardiomyocytes models may behave differently from each other, we evaluated the flexibility of our method by assessing chronic cardiotoxicity on a different cell line (Cor.4U cardiomyocytes). We performed repeated administration of dofetilide (100 nM) and nifedipine (60 nM) for three days and evaluated the responses to the drugs over time. Dofetilide is a strong hERG potassium channel blocker associated to a high torsade risk and significant QT prolongation (Naccarelli et al. 2003; Redfern et al. 2003; Kramer et al. 2013), whereas nifedipine is a L-type calcium channel blocker, known to decrease the AP duration (Guo et al. 2011; Harris et al. 2013; Huo et al. 2017). Our results show, as expected, that dofetilide increases the APD of Cor.4U cells whereas nifedipine decreases the APD in each DIV of administration (Supplementary Fig. S5 a, c). After each measurement, the drug was washed out and administrated again the day after. The analysis of APD50 and APD90, expressed as percentage of variation in respect to the control without drug, reports the drug effect over different days (Supplementary Fig. S5 b, d).

### Long-term effects of doxorubicin on cardiomyocytes

**Fig. 5:**
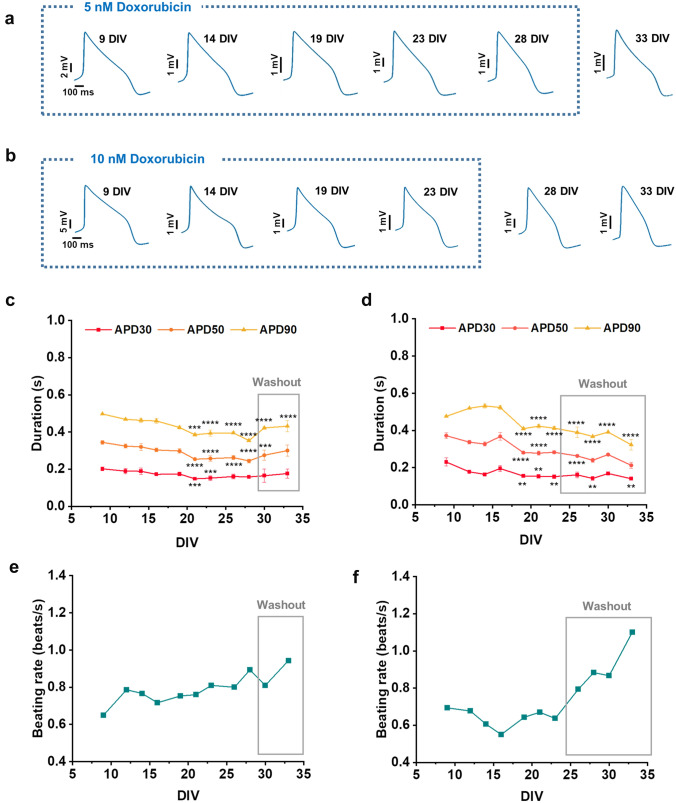
Long-term effect of doxorubicin on hiPSC-CMs. **a, b** Action potential mean waveforms at different days after 5 and 10 nM doxorubicin administration, respectively. **c, d** Action potential duration at different amplitudes (APD30, 50, 90) after 5 and 10 nM doxorubicin administration, respectively. **e, f** Beating rate after 5 and 10 nM doxorubicin administration in different wells. Data are represented as mean ± SEM of 3 wells

We have also explored the advantages of our approach in detecting long-term effects of doxorubicin on cardiac ion channels. Doxorubicin is an anthracycline antibiotic widely used in the treatment of several cancers (Todaro et al. 2013). However, doxorubicin causes chronic cardiotoxicity that can arise as late as 10 years after last administration, making extremely challenging to predict it during acute preclinical studies (Franco and Lipshultz 2015; Dong and Chen 2018). Therefore, to predict the long-term cardiotoxicity of this kind of drug, it is necessary to increase the assessment time window. Currently, the long-term doxorubicin cardiotoxicity has been explored by endpoint assays using xCELLigence Real Time Cell Analyser (ACEA Biosciences) to evaluate cytotoxicity and beating frequency of hiPSC-CMs and the transcriptomic analysis to identify genomic biomarkers for anthracycline-induced cardiotoxicity (Chaudhari et al. 2016). Furthermore, chronic doxorubicin-induced cardiotoxicity was also assessed using the standard cell viability assay (MTT 3-(4,5-dimethylthiazol-2-yl)-2,5-diphenyltetrazolium bromide) (Karhu et al. 2020), investigating the metabolite signatures (Chaudhari et al. 2017), the contractile motion properties and cardiac biomarker development (Kopljar et al. 2017). However, current approaches for assessment of chronic doxorubicin-induced cardiotoxicity are based on endpoints assays and do not allow for a continuous monitoring of transmembrane ion currents to evaluate their role in delayed cardiac adverse effects. On the contrary, optoporation on transparent MEAs enables the monitoring of APs of the same hiPSC syncytia up to 24 DIVs (from 9 to 33 DIVs) after repeated doxorubicin exposure and the evaluation of cell recovery after drug washout. Figure 5a, b depicts APs waveforms along the entire 24 DIVs experiment at the two different concentrations (5 and 10 nM). We observed a decrease in spike amplitude for both tested concentrations (Fig. 5a, b), and a slight decrease of APD starting at 21 DIVs for the lower tested concentration and at 19 DIVs for the higher concentration (Fig. 5c, d).

We also report in Supplementary Fig. S6 the APD90 data expressed as percentage of variation in respect to the control. We observe a reduction of beating rate over time only at the higher concentration (Fig. 5e, f). However, further studies where electrophysiology is combined with other techniques, such as viability and morphological analyses, are needed for assessing comprehensively the cardiotoxicity of doxorubicin. Lastly, we did not observe arrhythmia events in our experiments with doxorubicin, likely because of the low concentration in respect to other scientific articles (Maillet et al. 2016). We observed no recovery from doxorubicin effects after drug washout. Our electrophysiological data are comparable with Maillet et al. studies, in which they observed both acute and chronic effects of doxorubicin in hiPSC-CMs, including decrease in spike amplitude, increase in beat rate and shortening in corrected field potential duration (cFPD) after 20 h of treatment, although using concentrations of doxorubicin in micromolar range (Maillet et al. 2016). Even though the electrical activity of hiPSC-CMs is usually affected only by concentrations of doxorubicin in the micromolar range, nanomolar concentrations are already enough to affect cell viability and cause mitochondrial alterations (Louisse et al. 2017). Moreover, arrhythmic beating activity and decreased beating amplitude were also reported upon a 6 day exposure to doxorubicin (150 nM), possibly correlated to a doxorubicin-induced decrease in cell number or structural cardiomyocyte changes (Chaudhari et al. 2016). In this context, our results extend the time window of assessment of long-term cardiotoxicity cause by doxorubicin by evaluating an accurate functional parameter such as the action potential, thus improving the understanding of the complex mechanisms underlying anthracycline cardiotoxicity.

## Discussion

In this work, we detect the effects of compounds to the APs of hiPSC-CMs and we demonstrate long-term cardiotoxicity assessment capabilities by performing optoporation and intracellular AP recordings for up to 35 DIVs on the same cardiac cells. We obtained data on multiwell MEAs with transparent electrodes from large hiPSC-CM syncytia that represent excellent biological models for cardiotoxicity assessments. The investigated duration on the same biological samples is substantially longer than what standard screening technologies offer. Hence, the approach promises to improve drastically the detection of chronic toxic effects of drug candidates.

Our analyses on APD changes over time among different culture wells highlight the risks of using independent syncytia as reference for dose–response measurements, because cardiac cell cultures can present substantial differences in long-term experiments. Hence, the possibility to follow changes of transmembrane ion currents over long-time on the same cells or same syncytium represents an efficient way to increase the reliability of the tests.

Our approach not only enables the accurate electrophysiological assessment of hiPSC-CMs over long time, but it also offers the possibility to monitor changes in cardiac transmembrane ion currents of single cells over time as long as the cells do not migrate from the electrode. Considering the impact of the cardiac model choice and the maturation state of hiPSC-CMs on drug compound responsiveness (da Rocha et al. 2017; Karhu et al. 2020), optoporation may significantly contribute to establish an appropriate experimental model for delayed toxicity screening in early drug development.

In details, we correctly detected for 11 DIVs (from 14 to 25 DIVs) alterations of cardiac transmembrane ion currents due to the chronic administration of pentamidine, an hERG trafficking inhibitor which only induces cardiotoxic effects after longer term exposure (Kuryshev et al. 2005; Obergrussberger et al. 2016; Asahi et al. 2019). We observed the prolongation of APD after long-term exposition to pentamidine and the ability of cardiomyocytes to recover after drug washout. With pentamidine, we were also able to detect arrhythmia effects such as EADs that are normally difficult to observe with standard MEA recordings. Furthermore, since cardiotoxicity is one of the major issues of anti-cancer therapy, we also investigated the capabilities of optoporation to assess the effects of long-term administration of doxorubicin, the most widely used chemotherapeutic drug for cancer therapy (Sritharan and Sivalingam 2021). We monitored APs of the same hiPSC syncytia up to 24 DIVs (from 9 to 33 DIVs) after repeated doxorubicin exposure finding a decrease in spike amplitude for both tested concentrations and an increase in beating rate only for the lower concentration. We found a decrease in APD90 at several DIVs for both tested concentrations. Moreover, we observed that cardiomyocytes were not able to recover from doxorubicin after washout.

Taken together, our results demonstrate that optoporation may facilitate the development of more reliable cardiac cellular models intended for therapeutic and diagnostic applications thanks to the ability to monitor hiPSC-CMs maturation in terms of transmembrane ion currents. Furthermore, our method may be an effective in vitro strategy to assess the long-term exposure of hiPSC-CMs to compounds and delayed drug-induced cardiotoxicity in general, drastically reducing the attrition rates and market withdrawals during the drug development process.

## Supplementary Information

Below is the link to the electronic supplementary material.

Supplementary file1 (PDF 609 KB)

## Data Availability

The datasets generated during and/or analysed during the current study are available from the corresponding author on reasonable request.
