# Supplementary material for: Long-term in vitro recording of cardiac action potentials on microelectrode arrays for chronic cardiotoxicity assessment
Source: Arch Toxicol. 2023 Jan 6;97(2):509–22. doi: 10.1007/s00204-022-03422-y (PMC9859891; doi:10.1007/s00204-022-03422-y)
Supplement: Supplementary file 1 — Supplementary file1 (PDF 609 KB) [file 204_2022_3422_MOESM1_ESM.pdf]

Supplementary Material

Long-term in-vitro Recording of Cardiac Action Potentials on Microelectrode Arrays for Chronic Cardiotoxicity Assessment

Giuseppina Iachetta [1], Giovanni Melle [2], Nicolò Colistra [2], Francesco Tantussi [1], Francesco De Angelis [1]*, Michele Dipalo [1,2]*

S1. Live/Dead assay after repeated optoporation

S2. Control with dimethyl sulfoxide (DMSO)

S3. Effects of drugs detachable using optoporation

S4. Long-term effect of pentamidine

S5. Repeated drugs exposures in Cor.4U cardiomyocytes

S6. Long-term effect of doxorubicin

S7. Data distribution in control condition

S1. Live/Dead assay after repeated optoporation

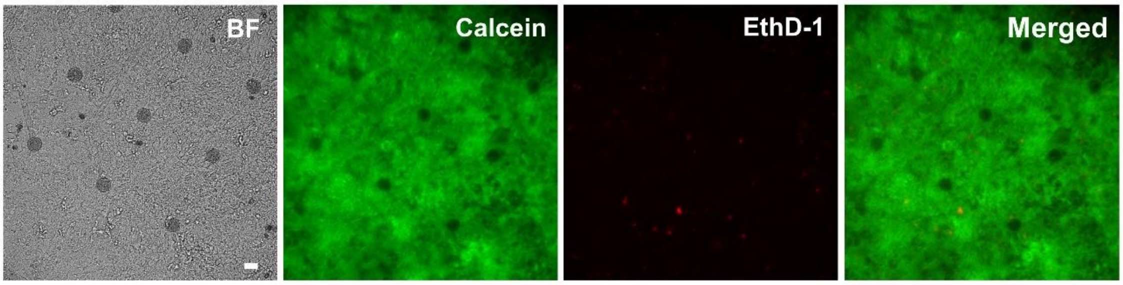

Fig. S1: **Live/Dead assay of cardiomyocytes on 6-well MEA (60-6wellMEA200/30iR-Ti-rcr) imaged with an inverted microscope (20X).** Live cells are stained with green fluorescence (Calcein AM), whereas dead cells with red fluorescence (Ethidium homodimer-1). The uniform syncytium of cardiomyocytes labeled with calcein AM confirms that the cells are healthy after 6 repeated opotoporation procedures. Scale bar: 30 μm.

S2. Control with dimethyl sulfoxide (DMSO)

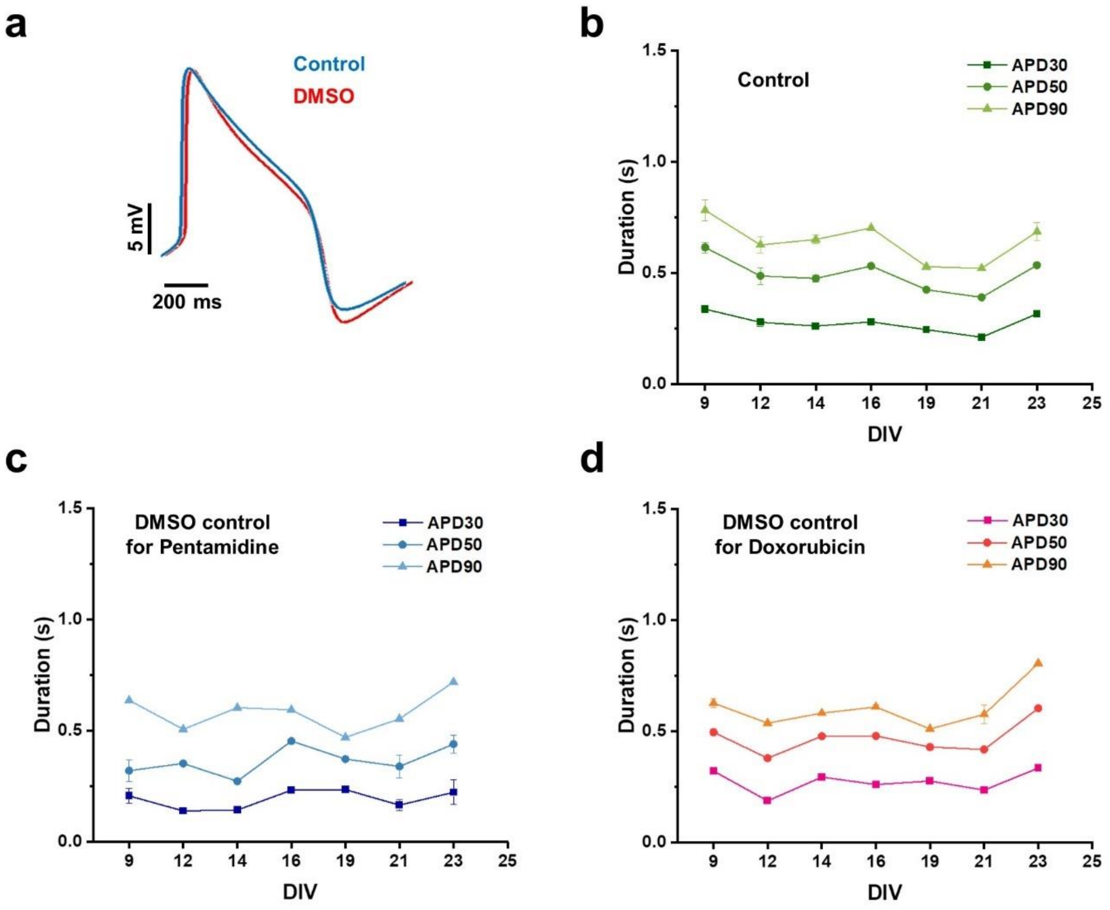

Fig. S2: Long-term effect of DMSO on hiPSC-CMs. (**a**) Intracellular action potential waveforms before (control) and after administration of DMSO on iCell cardiomyocytes. (**b**) Action potential duration (APD30, 50, 90) without DMSO (control). (**c, d**) Action potential duration (APD30, 50, 90) after the administration of the amount of DMSO used for 1.5 µM pentamidine and 10 nM doxorubicin administration, respectively. Data show that the administration of DMSO at tested concentration has no effect on the action potentials recorded with laser optoacoustic poration. Data are represented as mean ± SEM of 3 wells.

S3. Effects of drugs detachable using optoporation

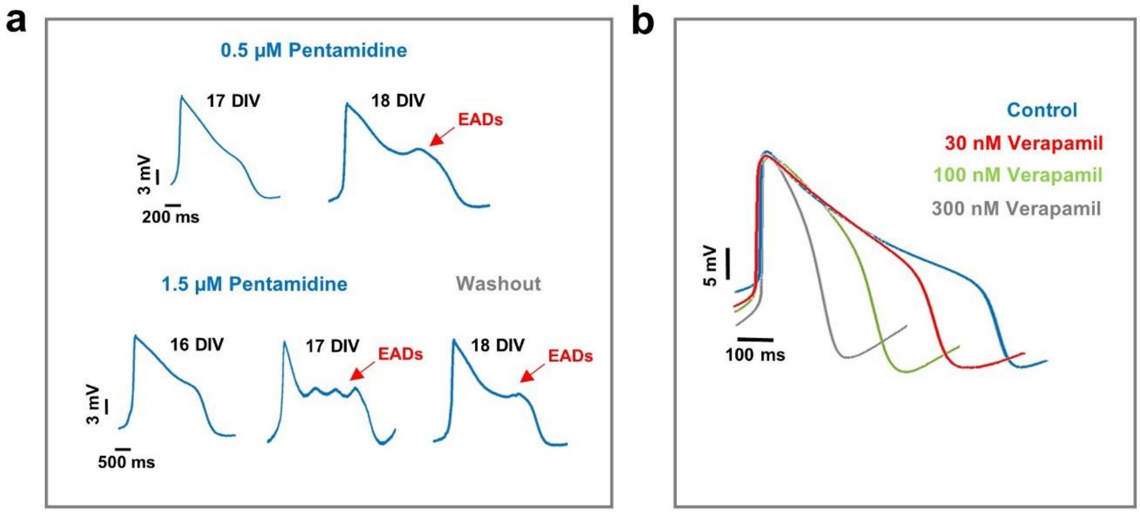

Fig. S3: (**a**) **Early afterdepolarization (EADs)** after 0.5 µM and 1.5 µM pentamidine administration, respectively. (**b**) **Action potential shape variation and triangulation** (e.g. after verapamil administration).

S4. Long-term effect of pentamidine

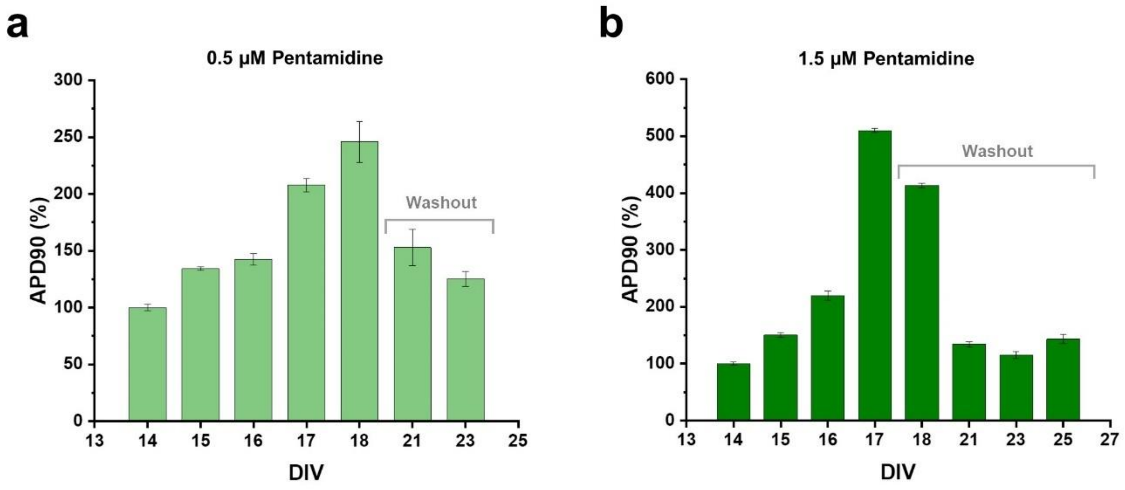

Fig. S4: Data analysis of long-term effect of pentamidine on hiPSC-CMs expressed as percentage of variation respect to control (100%). (**a, b**) Action potential duration at 90% of repolarization (APD90) after 0.5 µM and 1.5 µM pentamidine administration, respectively. Values are generated from the experiments showed in Fig. 4 and are represented as mean ± SD of 3 wells.

S5. Repeated drugs exposures in Cor.4U cardiomyocytes

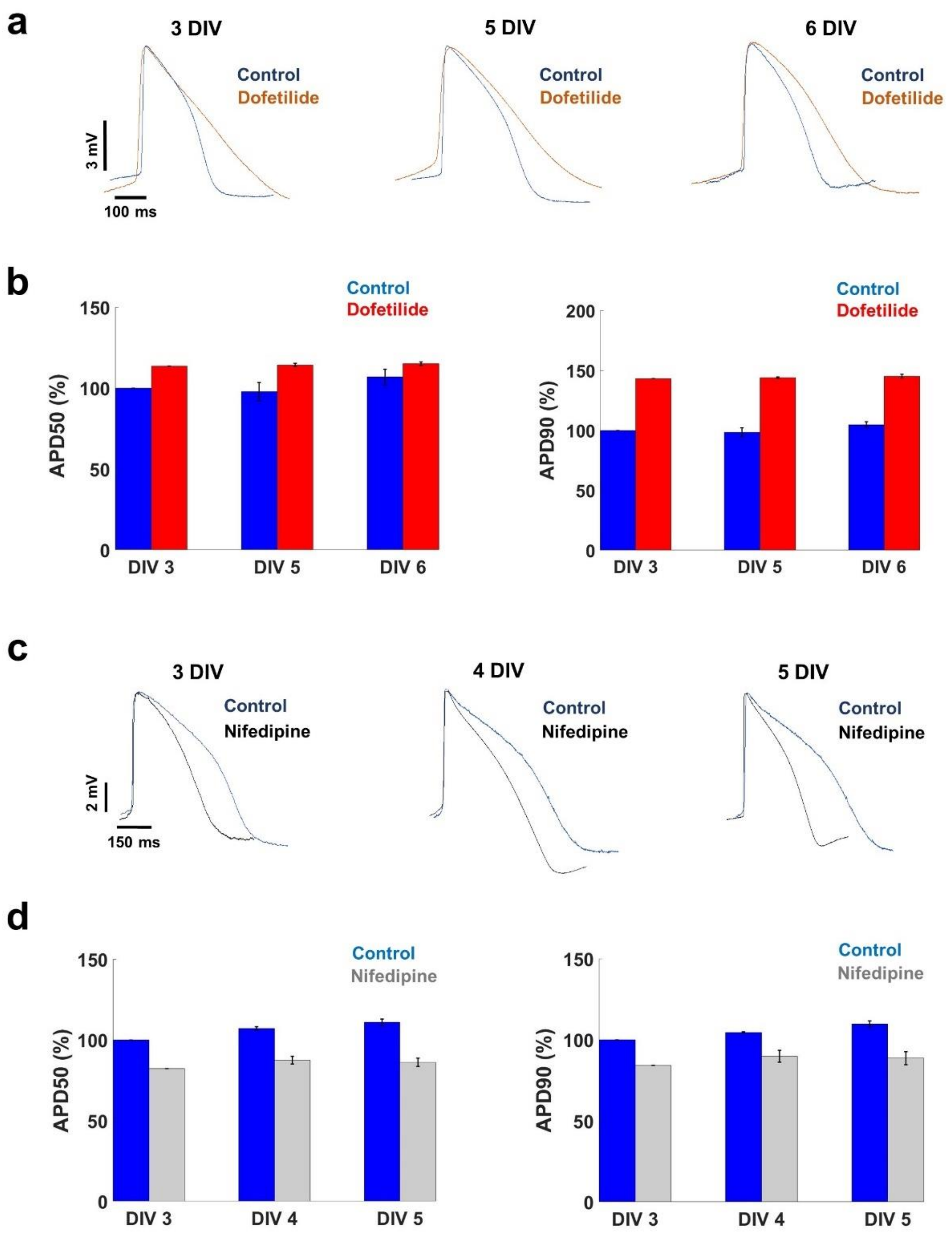

Fig. S5: Effects of dofetilide and nifedipine repeated administration on Cor.4U cardiomyocytes. (**a, c**) Action potential mean waveforms at different DIVs after administration of dofetilide (100 nM) and nifedipine (60 nM), respectively. (**b, d**) Action potential duration (APD50, 90) after dofetilide and nifedipine administration, respectively. Data are represented as mean ± SEM of 3 wells.

S6. Long-term effect of doxorubicin

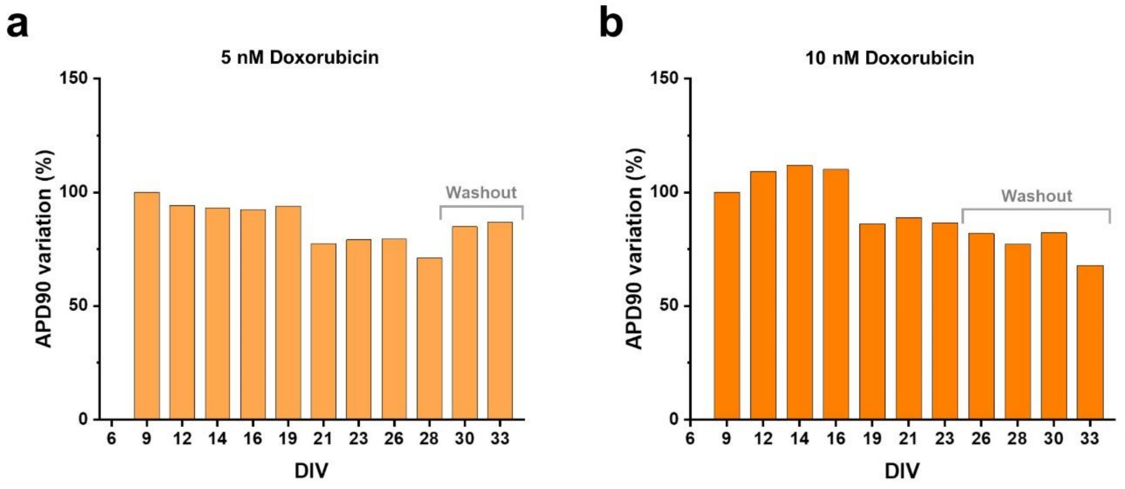

Fig. S6: Data analysis of long-term effect of doxorubicin on hiPSC-CMs expressed as percentage of variation respect to control (100%). (**a, b**) Action potential duration at 90% of repolarization (APD90) after 5 nM and 10 nM doxorubicin administration, respectively. Values are generated from the experiments showed in Fig. 5 and are represented as mean ± SD of 3 wells.

S7. Data distribution in control condition

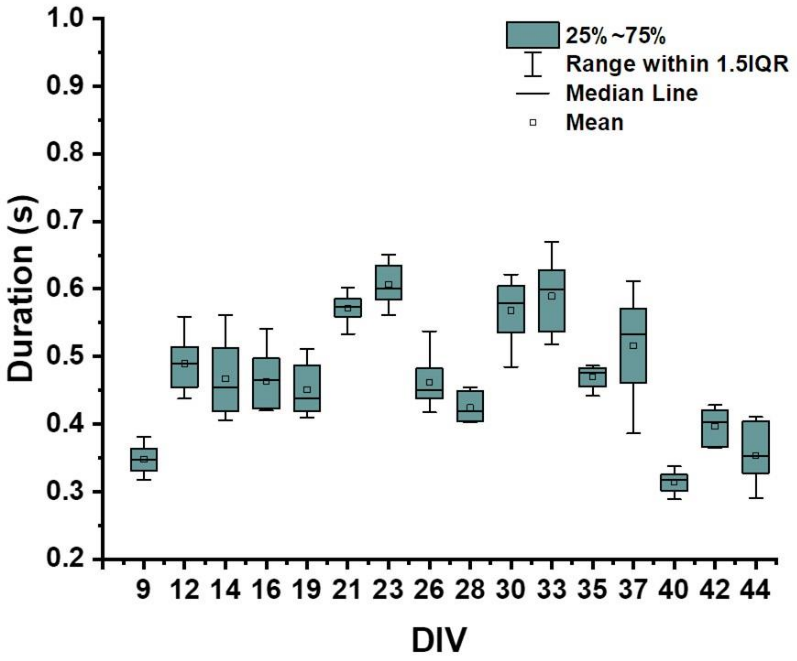

Fig: S7: Box plot of the action potential duration at 50% of the amplitude (APD50) in long-term measurements. The box plot graph shows the distribution of the data of APD50 for each day of measurement for the well of control reported in Fig. 2b.
